# Inactivation of *Pmel* Alters Melanosome Shape But Has Only a Subtle Effect on Visible Pigmentation

**DOI:** 10.1371/journal.pgen.1002285

**Published:** 2011-09-15

**Authors:** Anders R. Hellström, Brenda Watt, Shahrzad Shirazi Fard, Danièle Tenza, Paula Mannström, Kristina Narfström, Björn Ekesten, Shosuke Ito, Kazumasa Wakamatsu, Jimmy Larsson, Mats Ulfendahl, Klas Kullander, Graça Raposo, Susanne Kerje, Finn Hallböök, Michael S. Marks, Leif Andersson

**Affiliations:** 1 Science for Life Laboratory, Department of Medical Biochemistry and Microbiology, Uppsala University, Uppsala, Sweden; 2 Department of Pathology and Laboratory Medicine, Department of Physiology, and Cell and Molecular Biology Graduate Group, University of Pennsylvania, Philadelphia, Pennsylvania, United States of America; 3 Department of Neuroscience, Uppsala University, Uppsala, Sweden; 4 Institut Curie, Centre de Recherche, CNRS, UMR144, Structure and Membrane Compartments, PICT IBiSA, Paris, France; 5 Center for Hearing and Communication Research and Department of Clinical Neuroscience, Intervention, and Technology, Karolinska Institutet, Stockholm, Sweden; 6 Department of Veterinary Medicine and Surgery, College of Veterinary Medicine, University of Missouri–Columbia, Columbia, Missouri, United States of America; 7 Department of Clinical Sciences, Swedish University of Agricultural Sciences, Uppsala, Sweden; 8 Department of Chemistry, Fujita Health University School of Health Sciences, Toyoake, Japan; 9 Department of Immunology, Genetics, and Pathology, Uppsala University, Uppsala, Sweden; 10 Department of Animal Breeding and Genetics, Swedish University of Agricultural Sciences, Uppsala, Sweden; Medical Research Council Human Genetics Unit, United Kingdom

## Abstract

PMEL is an amyloidogenic protein that appears to be exclusively expressed in pigment cells and forms intralumenal fibrils within early stage melanosomes upon which eumelanins deposit in later stages. PMEL is well conserved among vertebrates, and allelic variants in several species are associated with reduced levels of eumelanin in epidermal tissues. However, in most of these cases it is not clear whether the allelic variants reflect gain-of-function or loss-of-function, and no complete PMEL loss-of-function has been reported in a mammal. Here, we have created a mouse line in which the *Pmel* gene has been inactivated (*Pmel* ^−/−^). These mice are fully viable, fertile, and display no obvious developmental defects. Melanosomes within *Pmel* ^−/−^ melanocytes are spherical in contrast to the oblong shape present in wild-type animals. This feature was documented in primary cultures of skin-derived melanocytes as well as in retinal pigment epithelium cells and in uveal melanocytes. Inactivation of *Pmel* has only a mild effect on the coat color phenotype in four different genetic backgrounds, with the clearest effect in mice also carrying the *brown/Tyrp1* mutation. This phenotype, which is similar to that observed with the spontaneous *silver* mutation in mice, strongly suggests that other previously described alleles in vertebrates with more striking effects on pigmentation are dominant-negative mutations. Despite a mild effect on visible pigmentation, inactivation of *Pmel* led to a substantial reduction in eumelanin content in hair, which demonstrates that PMEL has a critical role for maintaining efficient epidermal pigmentation.

## Author Summary

Pigmentation has since long constituted a prime model to study how genes act and interact. The *PMEL* gene encodes a protein exclusively found in the melanosomes of pigment-producing cells. Mutations in PMEL underlie some spectacular color phenotypes in animals including Dominant white color in chickens, Silver in horses, and Merle in dogs, but no spontaneous mutation causing a complete inactivation of this gene has yet been found in mammals. We have now developed a PMEL knockout mouse to further study the function of this protein. We show that mice lacking PMEL have almost normal visible pigmentation. However, loss of PMEL has a dramatic effect on the morphology of the melanosomes in skin, hair, and eye, such that the normally rod-shaped melanosomes in wild-type animals are spherical in the knockout mice. The knockout animals also have a substantial reduction in the content of black pigment in hair. The study establishes that PMEL has a critical role for maintaining normal pigment production.

## Introduction

Vertebrates produce two types of pigment - red/yellow pheomelanins and black/brown eumelanins [1]. Premelanosome protein (PMEL also known as PMEL17, SILV or gp100) is an integral membrane protein exclusively expressed in pigment cells that synthesize primarily eumelanins [2]. In mouse, PMEL expression starts at E9.5 in the presumptive retinal pigment epithelium (RPE) and at E10.5 in neural crest-derived melanoblasts, suggesting a function in the early stages of melanosome biogenesis [3]. Several studies have shown that fragments derived from proteolytic maturation of PMEL form the fibrillar matrix within melanosomes upon which eumelanins are ultimately deposited [4]. A role for PMEL fibrils in melanosome maturation is suggested by their ability to template and accelerate polymerization of highly reactive eumelanin precursors [5*]*–[**7]. The presence of PMEL has therefore been assumed to be critical for the normal production or stabilization of eumelanin but not pheomelanin. Fibrils formed by PMEL *in vitro* have biophysical hallmarks of amyloid such as those formed in Alzheimer's and Parkinson's diseases [6], providing a model for functional/non-pathological amyloid formation [8].

PMEL is well conserved among vertebrates and thus must play an important physiological role; the average amino acid sequence identity for PMEL among distantly related mammalian species (mouse-human) is in the range 75*–*80% (Table S1) and is as high as 50% between humans and zebrafish within certain subdomains [4]. Indeed, *PMEL* mutations resulting in hypopigmentation have been identified in a number of vertebrate species. The first reported mutation affecting PMEL function showed that the recessive *silver* allele in mouse (*Pmel^si^*) is caused by a single nucleotide insertion that truncates the last 25 C-terminal residues of PMEL within the cytoplasmic domain [9], [10]. This results in intracellular transport defects that deplete PMEL from early stage melanosomes [11]. Based on this mutation, the gene encoding PMEL was named *Silver* but has recently been renamed *Pmel* to provide a consistent nomenclature across species; the human homolog is named *PMEL* accordingly. The *silver* allele dilutes black/brown eumelanin but has no observable effect on pheomelanin and causes hair graying over time on black backgrounds [12], [13]. Microscopic examination of hairs revealed that some have no pigment at all, others had a reduced number of scattered pigment granules, while some were white with sparsely pigmented areas [12]. The graying phenotype was more pronounced in *silver* mice with mutations at the *Tyrosinase-related protein 1* locus (*Tyrp1* or *B*), indicating a gene interaction between *Pmel* and *Tyrp1* [13], [14]. Surprisingly the graying seemed to decrease with age in agouti mice, as opposed to the progressive graying on black backgrounds [12]. Relative to the elongated melanosomes in eumelanin-producing melanocytes from wild-type mice, melanosomes in immortalized melanocytes derived from *silver* mice are larger and round when analyzed by electron microscopy [11].

Mutations in *Pmel* homologs causing pigmentation phenotypes within a number of species have now been identified. In the domesticated chicken, a three amino acid insertion in the transmembrane (TM) region is associated with the Dominant white phenotype, inhibiting the production of all eumelanin in plumage, skin and uveal melanocytes in the choroid layer of the eye, whereas the RPE is not affected [15], [16]. Earlier studies showed that Dominant white was associated with irregularly shaped and assembled melanosomes [17] and pigment cell death [18]. In fact, Brumbaugh and Lee [19] suggested already 1975 on the basis of ultrastructural studies that *Dominant white* encodes a structural component involved in premelanosome formation which has now been confirmed by the identification of PMEL as the associated gene product. A five-amino acid deletion in the TM region and a point mutation downstream of the TM are associated with the *Dun* allele which has a similar effect on pigmentation as *Dominant white* [16]. The Smoky plumage color phenotype arose in a flock of Dominant white birds where pigmentation is partially restored due to a four amino acid deletion in the Polycystic Kidney Disease repeat domain [16], a lumenal region thought to be partially responsible for both PMEL localization [20] and fibril formation [21]. In horses, a missense mutation in the cytoplasmic region of *PMEL* is associated with dilution of black pigment in the mane and tail [22]. The zebrafish mutant *fading vision* (*fdv*) exhibits hypopigmentation in RPE and body melanocytes and a defect in vision, and is caused by a premature stop codon [23]. The Merle coat color pattern in dogs, characterized by patches of diluted pigment, is associated with the insertion of a short interspersed element (SINE) in intron 10 and the affected merle dogs exhibit auditory and ophthalmologic abnormalities resembling human patients with Waardenburg syndrome [24]. In cattle, a coat color dilution phenotype is associated with a missense mutation G22R in the signal peptide of PMEL, but conclusive evidence that this is the causative mutation has not yet been reported [25]. Importantly, in none of these cases is it clear that the mutation is a complete loss of PMEL function.

Mutations in genes important for coat pigmentation in laboratory mice are easily detected in mutagenesis screens and to date, 169 genes affecting pigmentation have been identified [26] (URL: http://www.espcr.org/micemut). In some of these genes, hundreds of mutations have been reported; for example, 104 alleles have been identified in the *Tyrosinase* (*Tyr*) gene, most of them causing albinism [27]. It is therefore somewhat surprising that only one *Pmel* mutation has been identified in mice as PMEL evidently has an important role in the melanocyte. This indicates that either (*i*) PMEL has other yet unknown functions and that inactivating mutations are embryonically lethal, or (*ii*) that complete loss-of-function mutations at the *Pmel* locus have no or only mild visible effects on pigmentation that are not readily detected in mouse mutagenesis screens.

To address the role of a non-functional PMEL *in vivo*, we have generated a knockout mouse line. In this study, we report that mice homozygous for the inactivated *Pmel* allele on black backgrounds have spherical melanosomes and display a weak dilution of eumelanin, similar to the one present in *Pmel^si^* mice. The data show that inactivation of *PMEL* does not dramatically disrupt pigmentation *in vivo*, and that mutations in *Pmel* homologs that render a loss of pigmentation therefore possess dominant-negative activity. The latter is demonstrated in an accompanying paper showing that the *Dominant white* allele in chicken, as well as the *Silver* allele in horses, encode pathogenic forms of the PMEL amyloid [28].

## Results

### Generation and validation of *Pmel* knockout mice

**Figure 1:**
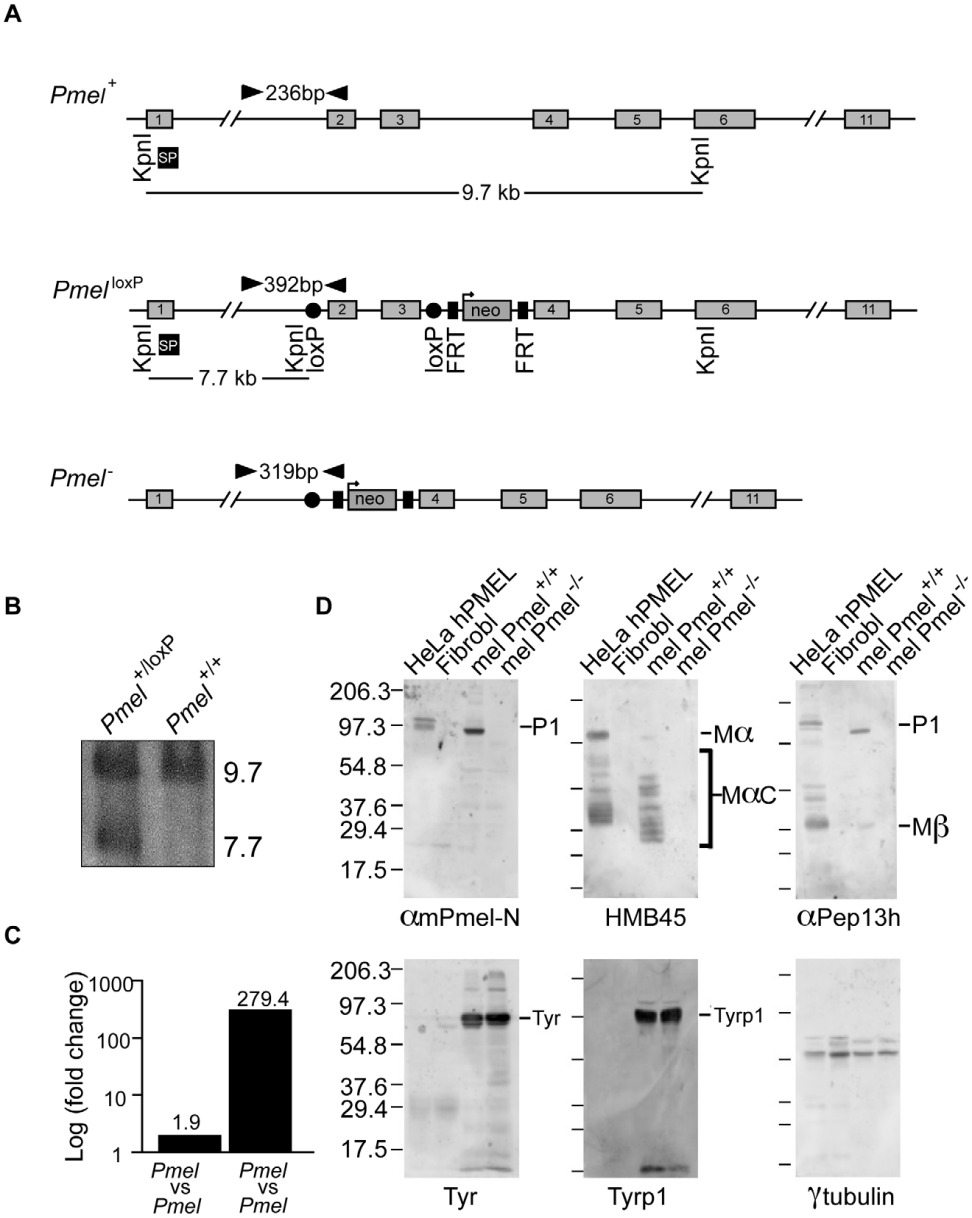
Strategy for the generation and validation of *Pmel^−/−^* mice. (A) Schematic representation of the *Pmel^+^*, *Pmel^loxP^*, and *Pmel^−^* alleles. The exons (gray boxes), primers used for genotyping (black arrowheads), KpnI restriction cleavage sites and the target site of Southern blot probe (SP, black box) are all indicated. In the *Pmel^loxP^* allele, exon 2 and exon 3 are loxP-flanked (red circles) and a neomycin (green box) gene is flanked by two FRT-sites (blue boxes). In the *Pmel^−^* allele, exon 2 and exon 3 are deleted after Cre-recombinase induced homologous recombination between the loxP sites. (B) Southern blot showing targeted and control ES-cells. The wild-type and the *Pmel^loxP^* alleles generate a 9.7 kb band and a 7.7 kb band, respectively. (C) *Pmel* mRNA expression. A 1.9-fold change was detected in skin tissue when homozygous *Pmel^+/+^* mice were compared to *Pmel* ^+/−^ heterozygotes. A 279-fold change was detected when homozygous *Pmel* ^−/−^ mice were compared to homozygous wild-type mice. (D) Western blot analysis. Whole cell lysates from primary cultures of skin-derived melanocytes from *wild-type* (mel Pmel^+/+^) or *Pmel* ^−/−^ (mel Pmel^−/−^) C57BL/6 mice were used, and lysates from transfected HeLa cells expressing human PMEL (HeLa hPMEL) or untransfected human fibroblasts (Fibrobl) were used as positive and negative controls. The lysates were fractionated by SDS-PAGE and analyzed by immunoblotting with a panel of antibodies directed against PMEL (αmPmel-N, HMB45, and αPep13h) or the control proteins, tyrosinase (TYR), tyrosinase-related protein 1 (TYRP1), and γ-tubulin. αmPmel-N recognizes an N-terminal peptide from mouse PMEL; HMB45 recognizes an epitope derived from the central region of PMEL that is enriched on fibrils; and αPep13h detects the PMEL C-terminus. Migration of molecular weight markers (in kDa) is shown to the left, and relevant bands are indicated to the right.

To generate a knockout construct, we prepared a pFlrt3-vector that included a fragment spanning exon 2 to exon 3 of the *Pmel* gene flanked by loxP sites and a neomycin selection cassette flanked by FRT sites (Figure 1A). The target construct was introduced into R1 ES-cells by electroporation and gene targeting was confirmed by long range PCR (data not shown) and Southern blot analysis (Figure 1B), in which the targeted gene generates a novel 7.7 kb band. Positive ES-cell clones were injected into blastocysts, which subsequently were implanted into female foster mice. Chimeric male offspring were bred to C57BL/6J mice and their offspring carrying the targeted *Pmel* allele were subsequently bred to PGK-Cre transgenic mice (C57BL/6J), in which Cre is driven by the ubiquitously expressed PGK promoter [29], to generate *Pmel* ^+/−^ offspring. The *Pmel* ^+/−^ mice were further bred to C57BL/6J for two subsequent generations, and *Pmel* ^+/−^ offspring were then intercrossed to generate homozygous null offspring. The loss of *Pmel* mRNA expression was confirmed by a qPCR test, using cDNA synthesized from skin RNA, that showed about two-fold reduction of the *Pmel* transcript in skin from *Pmel* ^+/−^ mice relative to *Pmel^+/+^* mice as expected, and a 279-fold reduction of the transcript level in *Pmel* ^−/−^ mice compared to *Pmel^+/+^* littermates (Figure 1C); the latter likely reflects the transcript detection limit by this assay, suggesting that *Pmel* ^−/−^ mice lack *Pmel* expression.

Primary cultures of skin-derived melanocytes were established from neonatal wild-type C57BL/6 and *Pmel* ^−/−^ mice. Immunoblotting analyses with a panel of three PMEL antibodies showed that PMEL protein was readily detected in whole cell lysates of wild-type melanocytes (mel Pmel^+/+^) but absent from lysates of *Pmel* ^−/−^ melanocytes (mel Pmel^−/−^) (Figure 1D, upper panels). Lysates from transfected HeLa cells expressing human PMEL (HeLa hPMEL) or untransfected human fibroblasts (Fibrobl) were used as positive and negative controls, respectively. By contrast, two melanocyte-specific proteins, tyrosinase (TYR) and tyrosinase-related protein 1 (TYRP1), and a house-keeping protein, γ-tubulin, were expressed at similar levels in wild-type and *Pmel* ^−/−^ melanocytes (Figure 1D, lower panels). Thus we conclude that our construct effectively inactivated the expression of PMEL in skin-derived melanocytes, but did not affect the expression of other melanogenic proteins.

### Inactivation of *Pmel* does not disrupt pigment synthesis but alters the shape of melanosomes

**Figure 2:**
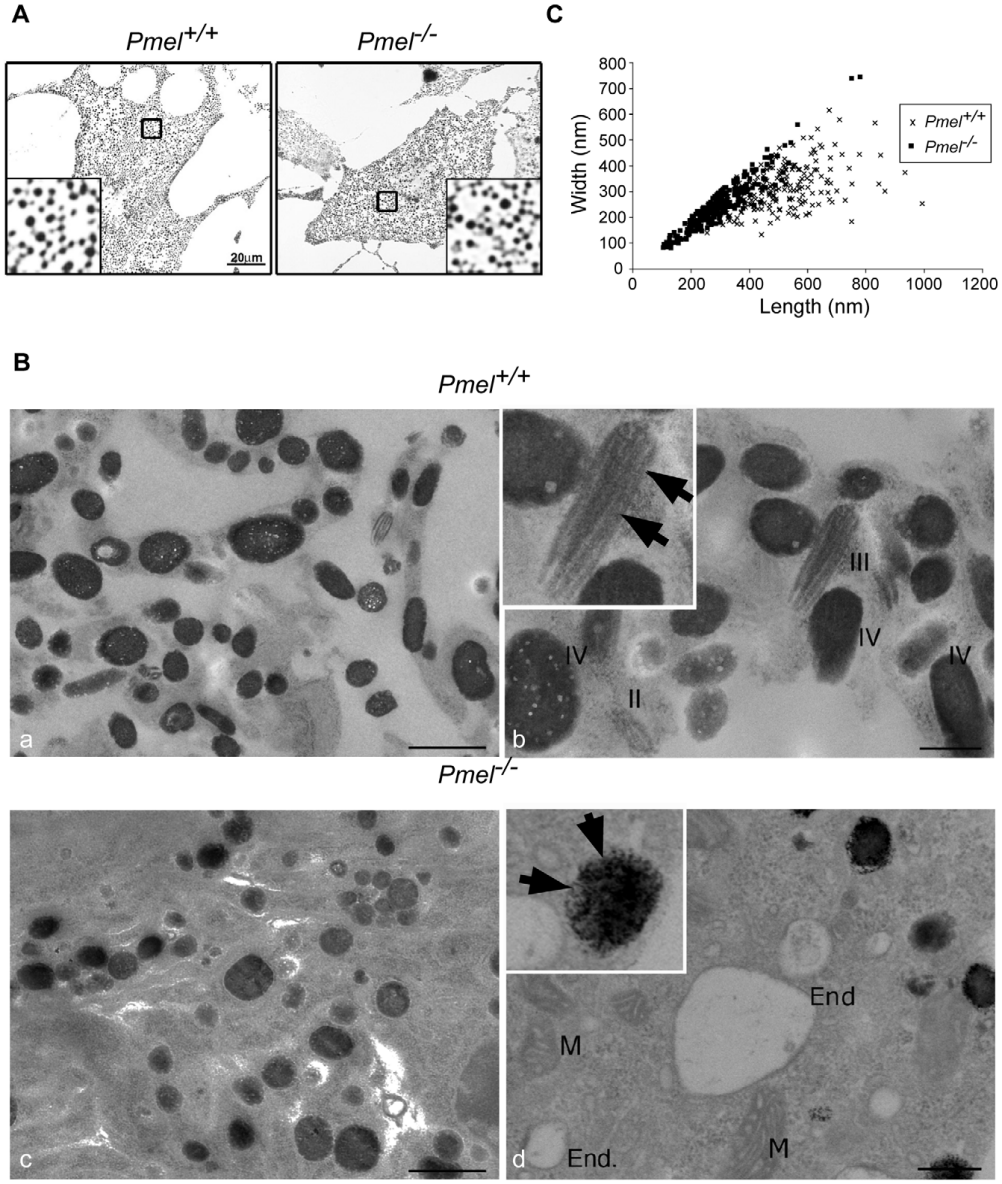
Morphology of melanosomes observed in primary cultures of skin-derived melanocytes from wild-type C57BL/6 (*Pmel^+/+^*) and *Pmel^−/−^*mice. (A) Bright-field microscopy; scale bar, 20µm; inset, 5X magnification. Note that the distribution of melanosomes is similar in both sets of melanocytes. (B) Electron microscopy. Cross-sections of melanosomes in the *Pmel^+/+^* melanocytes are both spherical and rod shaped, whilst they are only spherical in the *Pmel* ^−/−^ melanocytes. I, II, III, and IV represent stage I, II, III, and IV melanosomes. M, mitochondria; End, endosomes. Note the presence of fibrillar stage II and III melanosomes in the wild-type melanocytes (panel Bb, marked with arrowheads in inset) in contrast to the granular deposits of melanin in *Pmel* ^−/−^ melanosomes (panel Bd, marked with arrows in inset). Bars: 500 nm and 200 nm, in left and right panels respectively (C) Plot of the diameters of melanosomes along the long (length) and short (width) axes measured from electron microscopy images; n>200 for both genotypes. There was a weak correlation between the length and width of melanosomes in *Pmel^+/+^* cells (r = 0.52), consistent with their ellipsoidal shape, whereas in *Pmel* ^−/−^ cells, there was a strong correlation (r = 0.93), indicating a spherical shape. The difference in the length/width ratio between wild-type and *Pmel* ^−/−^ is overwhelmingly significant (Analysis of variance; F = 161, d.f.1 = 1, d.f.2 = 451; P<10^−6^).

To assess the requirement for PMEL in pigmentation and melanosome distribution, primary cultures of skin-derived melanocytes from wild-type C57BL/6 and *Pmel* ^−/−^ mice were analyzed by bright-field microscopy (Figure 2A). No obvious alteration in the degree of pigmentation could be observed in *Pmel* ^−/−^ primary melanocytes compared to the wild-type by this analysis. To better appreciate the effect of the loss of PMEL on melanosome ultrastructure, primary melanocytes from *Pmel^+/+^* and *Pmel* ^−/−^ mice were analyzed by electron microscopy. This analysis demonstrated that the loss of PMEL causes an altered shape of melanosomes (Figure 2B). Melanosomes in wild-type mice are rod-shaped; thus, in thin sections in which the rod-shaped structures are sectioned in random planes, the melanosomes appeared as a mixture of ellipsoids (transverse sections) and spheres (cross-section; Figure 2B). By contrast, melanosomes in *Pmel* ^−/−^ mice were always spherical, suggesting that they were no longer rod-shaped (Figure 2B). To quantify this effect, we measured the diameters of melanosomes along the long (length) and short (width) axes (Figure 2C). There was a weak correlation between the length and width of melanosomes in wild-type homozygotes (r = 0.52), consistent with their ellipsoidal shape, whereas in the knockout mice, the width and length of melanosomes were strongly correlated (r = 0.93), consistent with a spherical shape. Together, these data show that PMEL is not required for pigment production *per se*, but is necessary for the normal ellipsoidal shape of melanosomes.

The electron microscopy analysis revealed a number of additional features of melanosome architecture that were altered in *Pmel* ^−/−^ melanocytes relative to wild-type cells. First, whereas fibrillar stage II and III melanosomes were readily identified within wild-type melanocytes, no fibrillar intermediates were detected in *Pmel* ^−/−^ melanocytes although endosomal organelles were clearly visible (Figure 2B, compare Figure 2Bb and Figure 2Bd). This was expected given the previously established role for PMEL as the structural foundation for the fibrils. Second, many of the melanosomes in *Pmel* ^−/−^ melanocytes were associated with dense, granular deposits of melanin rather than the smooth, fibrillar deposits observed in wild-type melanocytes (Figure 2Bd). Together, these data suggest that PMEL is also required for the normal polymerization of melanin, with potential adverse consequences for melanosome integrity.

### PMEL is not required for melanosome maturation or segregation from late endosomes/lysosomes

**Figure 3:**
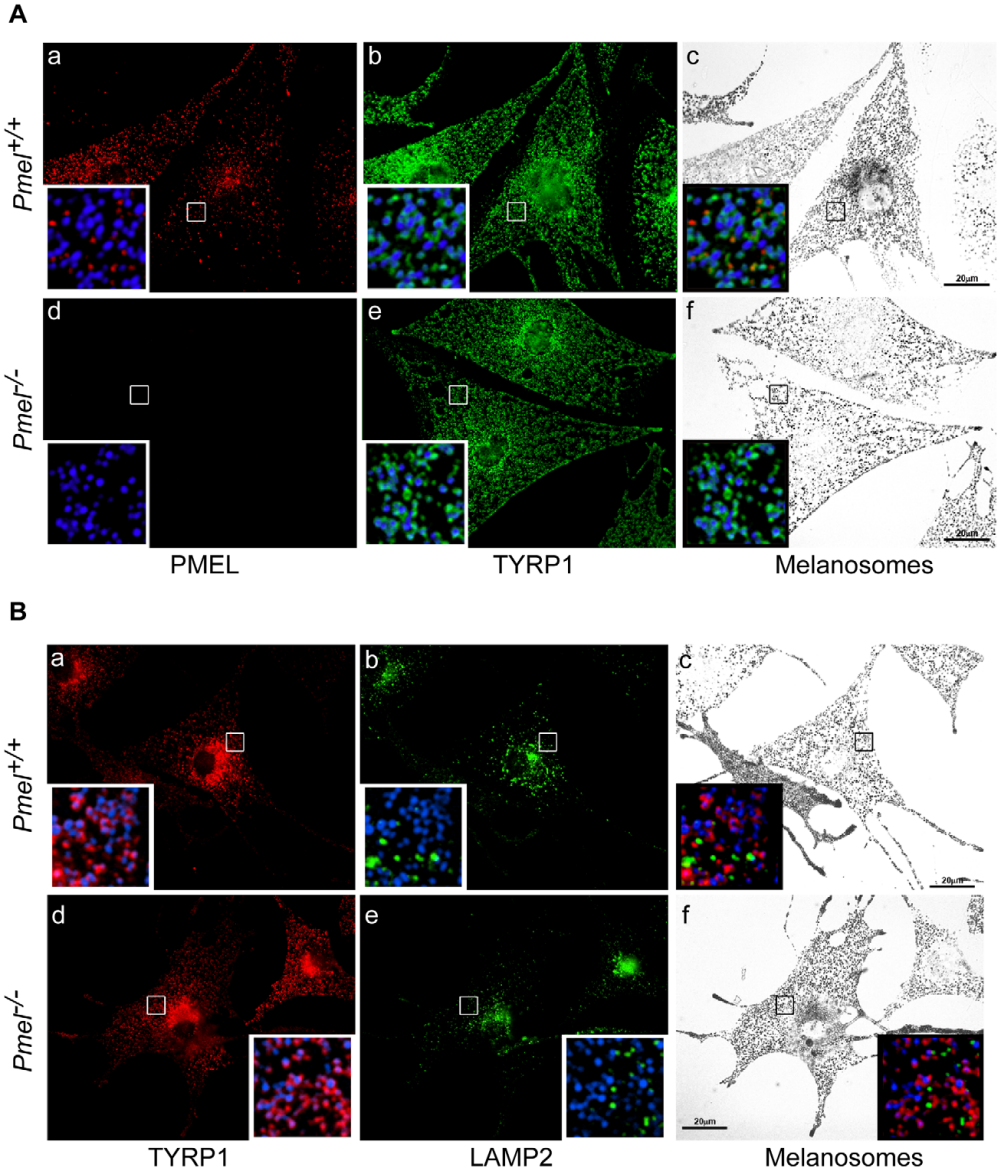
Immunofluorescence and bright field microscopy of primary melanocytes from C57BL/6 wild-type (*Pmel^+/+^*) and knockout (*Pmel* ^−/−^) mice. (A) Cells immunolabeled with antibodies against the mature PMEL protein (HMB45, red; *a*, *d*, insets) and TYRP1 (green; *b*, *e*, insets); the melanosomes were visualized by bright field (*c*, *f*) and pseudo-colored blue in the insets. Note the presence of TYRP1 surrounding melanosomes in both sets of melanocytes. (B) Cells immunolabeled with antibodies against the late endosome/ lysosome marker LAMP2 (green; *b*, *e*, insets) and TYRP1 (red; *a*, *d*, insets); the melanosomes were visualized by bright field (*c*, *f*) and pseudo-colored blue in the insets. Note the segregation of LAMP2 labeling from pigment granules labeled by TYRP1. Size bar, 20 µm.

PMEL fibrils in wild-type melanocytes begin to form in stage I melanosomes, which mature into stage II melanosomes by fibril assembly and into stage III/IV melanosomes following the delivery of membrane-bound melanosome cargoes and consequent melanogenesis [30], [31]. However, stage I melanosomes are also accessible to endocytic cargo and appear to be intermediates in the maturation of late endosomes and lysosomes, which are separate from later stage melanosomes [31]. We therefore investigated the possibility that PMEL is required for the normal development of melanosomes and the segregation of cargoes destined for late stage melanosomes from those destined for late endosomes/ lysosomes. Primary melanocytes from wild-type C57BL/6 and *Pmel* ^−/−^ mice were analyzed by immunofluorescence and bright field microscopy for markers of melanosomes and late endosomes/ lysosomes (Figure 3). As expected, *Pmel* ^−/−^ melanocytes lacked labeling for the PMEL-specific antibody HMB45 (Figure 3Aa, d) but were robustly labeled for TYRP1 (Figure 3Ab, e). Importantly, labeling for TYRP1 predominantly surrounded pigment granules in both wild-type and *Pmel* ^−/−^ melanocytes (Figure 3Ac, f and insets). Furthermore, both TYRP1 and pigment granules showed only minimal overlap with LAMP2, a marker specific for late endosomes/ lysosomes (Figure 3B; note insets in panels c, f). These data demonstrate that PMEL is not required for either melanosome maturation or for the segregation of melanosomes from late endocytic compartments.

### PMEL expression and function in skin, eye, and inner ear

**Figure 4:**
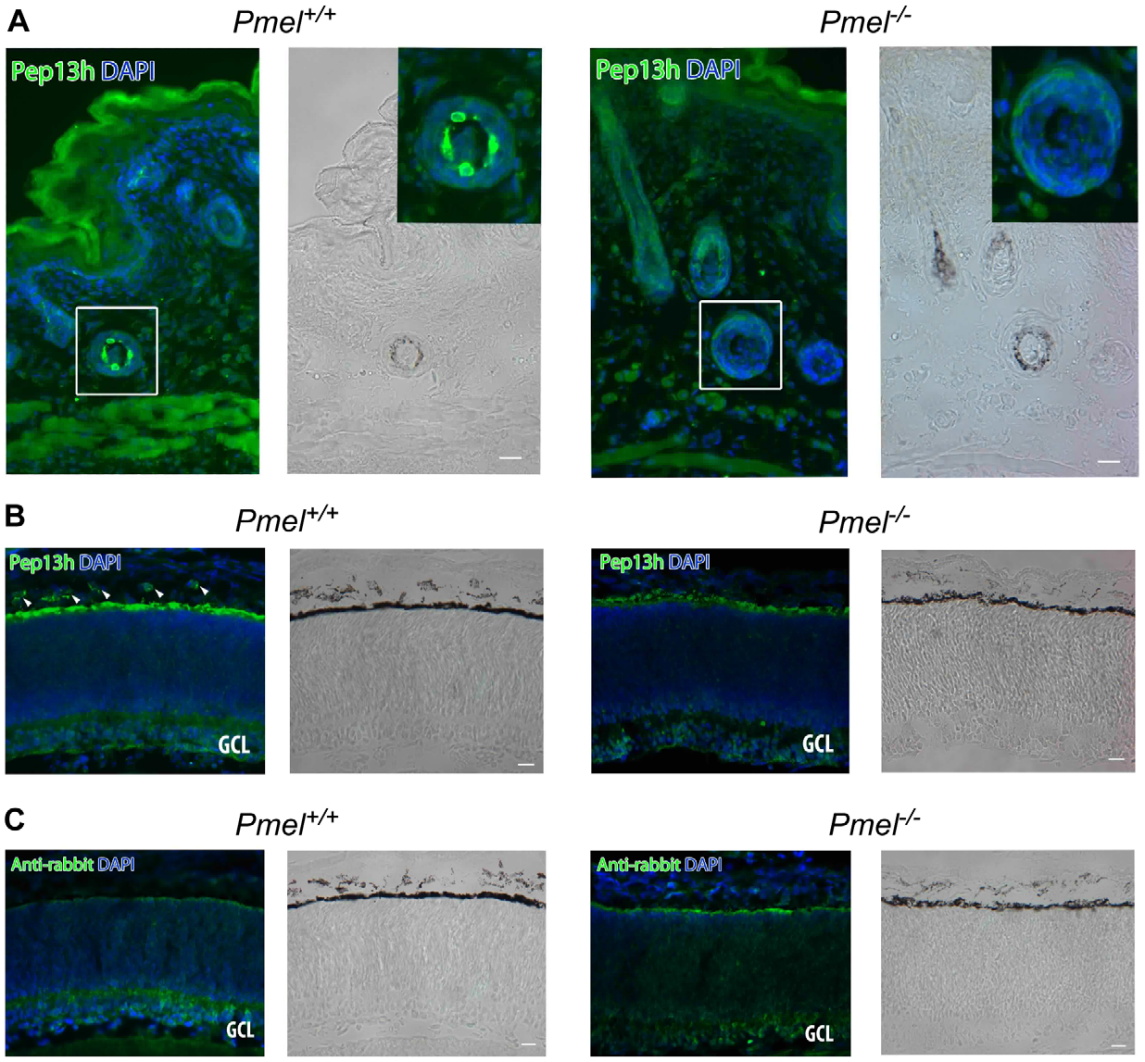
Immunohistochemistry of skin and retina from *Pmel^+/+^*and *Pmel^−/−^*mice. Sections of skin (A) or retina (B, C) from *Pmel^+/+^* (a, b) and *Pmel* ^−/−^ (c, d) mice were immunolabeled with αPep13h (anti-PMEL) (A, B) or secondary antibody alone (C) and counterstained with DAPI to label nuclei, and analyzed by immunofluorescence microscopy (a, c) or bright field microscopy to assess pigmentation (b, d). (A) PMEL-immunoreactive cells were found in the hair follicles of the skin in wild-type mice (*a–b*), but not in the *Pmel* ^−/−^ mice (*c–d*). Insets, two-fold enlargement of the boxed region. (B) αPep13h immunoreactive cells (green) were found in the choroid (arrowheads) and in the RPE from the *Pmel^+/+^* mouse (*a*), but only in the RPE/ Bruch's membrane from the *Pmel* ^−/−^ mouse (*c*). GCL, ganglion cell layer. (C) Immunostaining obtained using only the secondary anti-rabbit antibody shows a signal from Bruch's membrane. Size bar, 20 µm.

We investigated whether the inactivation of PMEL expression caused changes in morphology or pigmentation in skin, eye, and inner ear. Skin from *Pmel^+/+^* mice had pigmented cells in hair follicles that were immunoreactive for PMEL using the Pep13h antiserum (Figure 4Aa, b). The Pep13h antiserum detects the cytoplasmic domain only found on immature PMEL forms in early secretory organelles and the immunoreactivity thus reflects the site of PMEL synthesis [32]. Only background Pep13h-immunoreactivity was seen on pigmented cells in hair follicles in skin from the *Pmel* ^−/−^ mice (Figure 4Ac, d). The melanocytes in the knockout mice were intact and had pigmentation, thus PMEL is not required for pigmentation of melanocytes *in situ.*

Comparison of the choroid and outer layers of the retina in 3 day postnatal and 18-months old *Pmel^+/+^* and *Pmel* ^−/−^ mice, showed that the choroidal melanocytes and RPE cells were Pep13h-immunoreactive in *wild-type* but not the knockout mice (Figure 4B). Weak Pep13h-staining in the RPE of *Pmel* ^−/−^ mice was also seen in the secondary antibody-controls in both wild-type and knockout retinas, and thus represented background immunoreactivity likely due to the well-established non-specific binding to Bruch's membrane. Western blot analysis of retina from wild-type and knockout mice using the Pep13h-antiserum confirmed the absence of PMEL in the eyes from the *Pmel* ^−/−^ mice (Figure S1).

**Figure 5:**
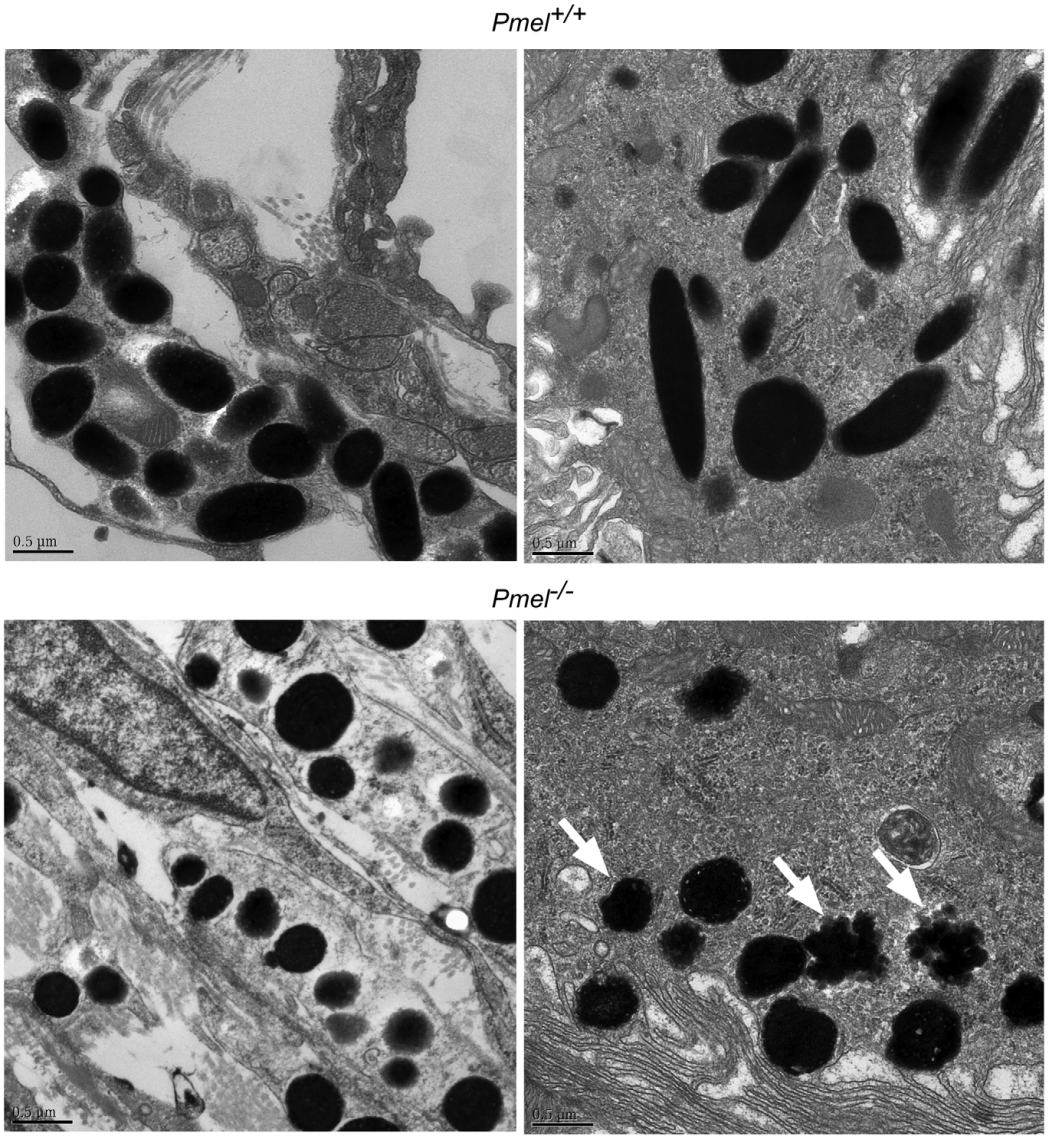
Electron microscopy of choroidal (upper and lower left micrographs) and RPE cells (upper and lower right micrographs), including melanosomes, from wild-type C57BL/6 mice (upper two micrographs) and *Pmel^−/−^* mice (lower two micrographs), respectively. A scale bar is depicted in the lower left hand corner of each micrograph. Melanosomes of both choroidal and RPE cells of the affected animal appear spherical with irregular borders, the latter more obvious in the RPE cells (lower right micrographs, indicated by white arrowheads). Further, no cigar-shaped melanosomes were found in either choroidal or RPE cells in the *Pmel* ^−/−^ mice.

To test whether melanosome shape was altered in eye pigment cells as in skin melanocytes, we analyzed thin sections of the retina by electron microscopy. Whereas melanosomes in the uveal melanocytes of the choroid layer and particularly in the RPE cells of wild-type mice were oblong, the melanosomes in both cell types in the *Pmel* ^−/−^ mice were spherical (Figure 5), in perfect agreement with the results obtained by electron microscopy of primary skin-derived melanocytes. Moreover, as in *Pmel* ^−/−^ skin melanocytes, many of the melanosomes in *Pmel* ^−/−^ RPE showed irregular melanin aggregates and poorly preserved membranes (Figure 5D). This demonstrates that PMEL is required for normal development of the melanosomes in all three cell types (skin melanocytes, choroid melanocytes, and RPE cells).

The striking changes in the structure of the RPE melanosomes suggested that the *Pmel* ^−/−^ mice might have alterations in retinal integrity that might impair their vision. To test this hypothesis, we performed electroretinography (ERG) on two 18-month old *Pmel* ^−/−^ mice and two age-matched controls. The results showed that amplitudes, implicit times, and waveforms for both dark-adapted and light-adapted responses were similar between *Pmel* ^−/−^ and *Pmel^+/+^* mice. Hence, the results showed that despite the changes in melanosome architecture, loss of PMEL expression did not cause a severe impairment of retinal function detectable with full-field ERG.

To test whether loss of PMEL expression affects the morphology of the inner ear, thin sections of cochlear samples dissected from the inner ears of two *Pmel* ^−/−^ mice and one wild-type mouse were analyzed by electron microscopy (Figure S2). All cells, including the intermediate cells harboring the pigment were represented and there was no obvious difference in overall morphology between the two genotypes. Furthermore, the knockout mice had normal Preyer reflexes, a rough physiological indication of “normal” hearing.

### Loss of PMEL affects coat and skin pigmentation in a qualitatively subtle but quantitatively substantial manner

**Figure 6:**
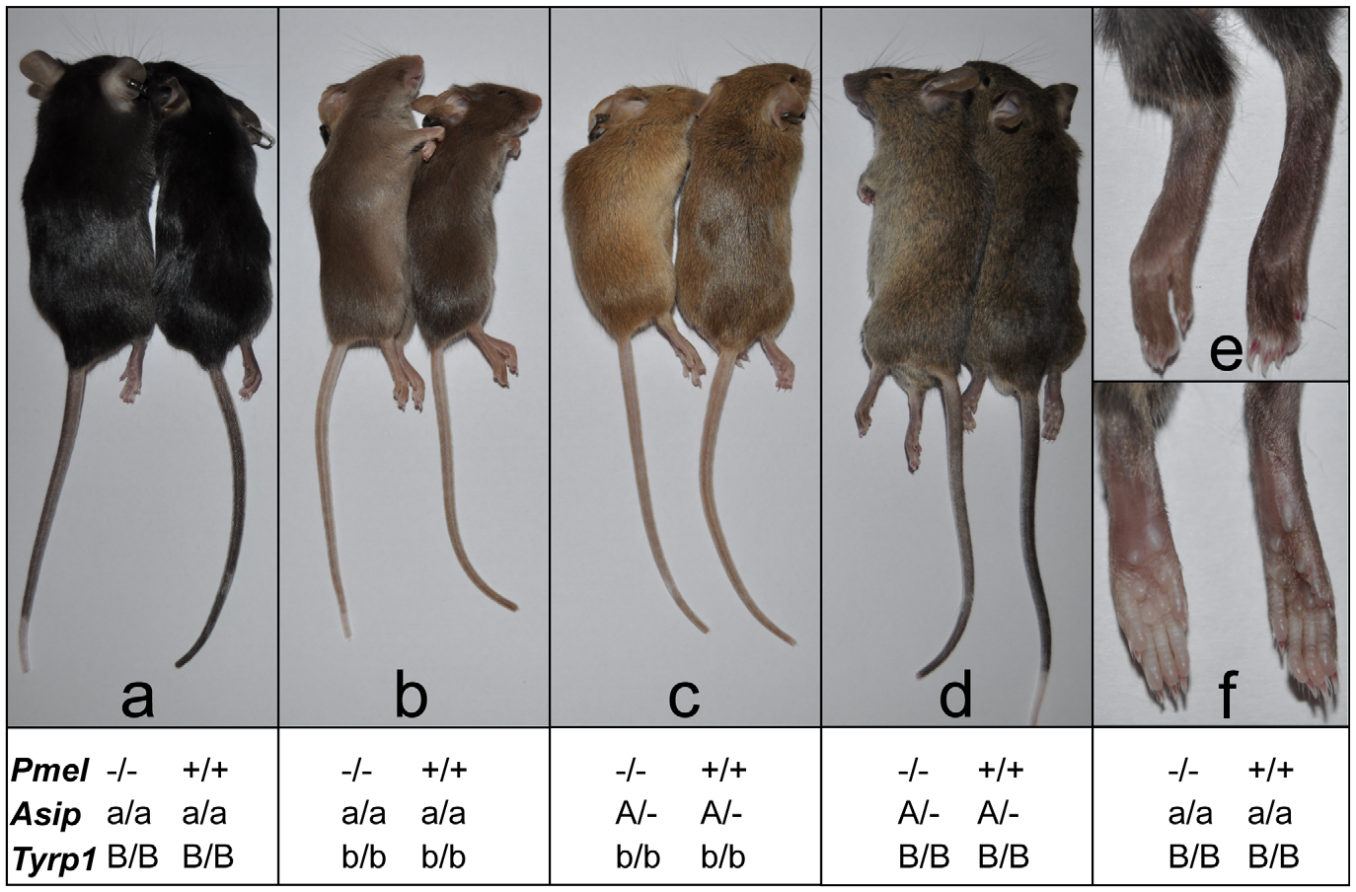
Phenotypic effects on pigmentation of the *Pmel^−/−^* genotype on four different genetic backgrounds. Coat color was assessed in different genetic backgrounds by pairwise comparisons from an F_2_ intercross generation. Within each pair, the *Pmel* ^−/−^ and *Pmel^+/+^* mice are to the left and right, respectively. (*a*) The non-agouti black (*Asip^a/a^*) *Pmel* ^−/−^ mice displayed a subtle dilution of coat color and tail skin. (*b*) PMEL inactivation in brown mice (*Asip^a/a^ Tyrp1^b/b^*) caused the most dramatic reduction in coat color and tail skin pigmentation. (*c*) The black hairs of the *Pmel* ^−/−^ mice on brown agouti (*Asip^A/−^ Tyrp1^b/b^*) background was diluted giving the coat a more yellow/light brown appearance compared to the wild-type. (*d*) The weakest phenotypic effect associated with the *Pmel* ^−/−^ genotype was seen in agouti mice (*Asip^A/−^*) but the coat and skin color were slightly lighter relative to the wild-type. (*e, f*) The skin of the feet of non-agouti black (*Asip^a/a^*) *Pmel* ^−/−^ mice were less pigmented compared to the wild-type.

The *Pmel* ^−/−^ mice are fully viable, fertile, and display no obvious developmental defects. Furthermore, *Pmel* ^−/−^ mice on a C57BL/6 background – which have the genotype *Asip^a/a^* at the locus encoding Agouti-signaling protein and thus make only eumelanin – did not display striking hypopigmentation. We therefore tested the effect of the *Pmel^-^* mutation on coat color dilution in different genetic backgrounds by intercrossing our C57BL/6 *Pmel* ^−/−^ line with BALB/c mice for three generations. In addition to segregating the *Pmel* locus, this intercross segregated three additional coat color loci: *agouti-signaling protein (Asip*), the classical Agouti (A) locus; *tyrosinase-related protein 1* (*Tyrp1*), the classical Brown (B) locus; and *Tyrosinase (Tyr*), the classical Albino (C) locus. The genotypes at these four loci in the parental lines of the intercross were *Pmel* ^−/−^, *Asip^a/a^*, *Tyrp1^B/B^*, *Tyr^C/C^* for the C57BL/6 line and *Pmel^+/+^*, *Asip^A/A^*, *Tyrp1^b/b^*, *Tyr^c/c^* for Balb/c (lower case letters represent the recessive allele at each locus). The *Pmel* knockout showed a subtle effect on black (*Asip^a/a^, Tyrp1^B/B^*; Figure 6a), brown (*Asip^a/a^, Tyrp1^b/b^*; Figure 6b), agouti (*Asip^A/A^, Tyrp1^B/B^*; Figure 6c), and brown agouti (*Asip^A/A^, Tyrp1^b/b^*; Figure 6d) backgrounds. On a black background, a weak silvering effect in the coat color was seen, and the pigmentation on the paws and tail was reduced (Figure 6e, f), suggesting perhaps a more pronounced effect on pigmentation in interfollicular epidermis and dermis. The most pronounced effect of the *Pmel* knockout was observed on the brown background, where the brown pigmentation was markedly diluted. Brown agouti, *Pmel* ^−/−^ mice had an apparent dilution of the dark hairs, whilst the yellow pigmentation seemed unaffected. A more subtle effect of the *Pmel* ^−/−^ genotype was noted in agouti mice, but their paler tails distinguished the knockouts from the wild-type littermates. Together, these data confirm a previously described genetic interaction between the *Pmel silver* (*Pmel^si/si^*) allele with the brown (*Tyrp1*) locus [13], [14]. However, a difference was that the previous studies indicated that *silver* showed a more pronounced effect in *Tyrp1* heterozygotes whereas we only observed an altered pigmentation in *Tyrp1^b/b^* homozygotes. The results of this cross-breeding experiment show that PMEL only modestly influences visible pigmentation in hair and skin.

The effect of inactivating *Pmel* on pigment production was also assessed quantitatively by a chemical analysis using previously described methods [33*]*–[**36]. A comparison of pigment content in hair from *wild-type* and *Pmel* ^−/−^ mice on a C57BL/6 background demonstrated that loss of PMEL expression causes a substantial reduction (−44%) of eumelanin content (Table 1); no significant effect on pheomelanin content was noted. This result was further corroborated by analyzing melanin content in the eight animals depicted in Figure 6. In each pairwise comparison on the same genetic background, the knockout mouse showed a lower amount of eumelanin in hair, ranging from 35*–*69% of the level observed in wild-type hair (Table 2). The most pronounced reduction was observed in animals on a *Tyrp1^b/b^* background, consistent with the visual appearance (Figure 6). No consistent difference in pheomelanin-content was noted among *Pmel* genotypes; by comparison, homozygosity for the *non-agouti* allele at the *Asip* locus caused a drastic reduction in pheomelanin content (Table 2).

**Table 1:** Content of total melanin, eumelanin, and pheomelanin in *wild-type* and *PMEL^−/−^* mice on a C57BL/6 background (*Asip^a/a^*).

	*PMEL* genotype			
Measure	*+/+* (n = 3)	−/− (n = 4)	t-test (d.f. = 5)	% of wild-type
A500/mg^a^	0.902±0.048	0.518±0.045	10.8***	
Total melanin (µg/mg)^a^	91.1	52.3		57.4
PTCA (ng/mg)^b^	4504±177	2528±168	15.1***	
Eumelanin (µg/mg)^b^	112.6	63.2		56.1
4-AHP (ng/mg)^c^	27.0±1.7	31.5±2.6	−2.5	
Pheomelanin (µg/mg)^c^	0.24	0.28		116.7

a Absorbance at 500 nm, a measure of total melanin after Soluene-350 solubilization [34]. Total melanin content was estimated as A500/mg multiplied by a factor of 101.

b Pyrrole-2,3,5-tricarboxylic acid (PTCA), a degradation product of eumelanin [33]. Eumelanin content was estimated by multiplying PTCA content by a factor of 25.

c 4-Amino-3-hydroxyphenylalanine (4-AHP), a degradation product of pheomelanin. Pheomelanin content was estimated by multiplying 4-AHP by a factor of 9 [36].

**Table 2:** Eumelanin and pheomelanin content in *wild-type* and *PMEL^−/−^* mice on mice on different genetic backgrounds.

Genotype	Phenotype	4-AHP (ng/mg)^a^	Pheomelanin (µg/mg)^a^	PTCA (ng/mg)^b^	Eumelanin (µg/mg)^b^	Eumelanin compared with *wild-type*
(*Pmel Asip Tyrp1*)						
*+/+ a/a B/B*	black	7.0	0.06	3087	77.2	
−/− *a/a B/B*		27.1	0.24	2126	53.2	68.9%
*+/+ a/a b/b*	brown	42.4	0.38	1222	30.6	
−/− *a/a b/b*		35.0	0.32	620	15.5	50.7%
*+/+ A/− B/B*	agouti	291	2.62	2635	65.9	
−/− *A/− B/B*		248	2.23	1491	37.3	56.6%
*+/+ A/− b/b*	brown agouti	414	3.73	985	24.6	
−/− *A/− b/b*		545	4.91	348	8.7	35.3%

The animals depicted in Figure 6 were analyzed.

a 4-Amino-3-hydroxyphenylalanine (4-AHP), a degradation product of pheomelanin. Pheomelanin content was estimated by multiplying 4-AHP by a factor of 9 [36]. Based on a single determination per animal.

b Pyrrole-2,3,5-tricarboxylic acid (PTCA), a degradation product of eumelanin [33]. Eumelanin content was estimated by multiplying PTCA content by a factor of 25. Based on duplicate assays from a single animal/genotype.

### The allele frequency distribution at the human *PMEL* locus is consistent with purifying selection

Given the reduced hair and skin pigmentation in Europeans it is conceivable that *PMEL* loss-of-function mutations have been tolerated or even under positive selection in this population. To address this question we exploited the low coverage resequencing data for 60 European, 59 African, and 60 Asian individuals that have recently been released by the 1000 Genomes Project [37]. The result clearly indicated that this is not the case and the data are consistent with purifying selection acting on the human *PMEL* locus (Table 3). In the European population only synonymous substitutions were found. In total, seven SNPs causing missense mutations were found, most of them in Africa, but the minor allele frequency at all loci were below 15%. Furthermore, in all three populations synonymous SNPs showed the highest degree of genetic diversity, a typical feature of a locus under purifying selection.

**Table 3:** Observed frequencies of the non-reference allele at the human *PMEL* locus on chromosome 12 in African (YRI), European (CEU), and Asian (CHJPT) populations.

Nucleotide position (bp)	Nt change	AA position	AA change	YRI	CEU	CHJPT
54,637,613	G/A	247	Synonymous	0	0.31	0.26
54,637,395	G/T	320	Pro/His	0	0	0.03
54,637,269	G/A	362	Ala/Val	0.01	0	0
54,637,267	G/A	363	Pro/Ser	0.04	0	0
54,637,244	C/G	370	Glu/Asp	0.06	0	0
54,637,014	G/A	447	Thr/Met	0.02	0	0
54,635,873	C/T	505	Gly/Ser	0.14	0	0
54,635,388	A/G	596	Val/Ala	0.14	0	0
54,634,295	G/A	652	Synonymous	0.53	0.84	0.84

Data generated by the 1000 genome project.

## Discussion

The present study provides a substantial advance in understanding the role of PMEL and of the PMEL-derived amyloid fibrillar melanosome matrix in pigment cell biology. The PMEL protein has generally been thought to be important for pigmentation since mutations in PMEL orthologs in several species are associated with substantial pigment dilution, in some cases attributable to poor melanocyte survival. This study demonstrates that PMEL is essential for the normal development of the rod-shaped melanosomes in eumelanin-producing melanocytes in hair, skin, and eye, but not for the general survival of pigment cells in several tissues. Thus, while the amyloid fibrils formed by proteolytic fragments of PMEL play a critical role in generating the characteristic rod-shape of melanosomes, the *Pmel* knockout mutation has a relatively mild effect on visible pigmentation. A characteristic difference between melanosomes in eumelanin- versus pheomelanin-producing melanocytes is the shape of the melanosomes, rod-shaped versus spherical. The present study demonstrates that the shape of the melanosomes is not critical for which type of pigment is produced. Moreover, despite a dramatic effect on melanosome shape and melanin deposition in the RPE and choroid, visual function in *Pmel* ^−/−^ mice is not substantially impaired. Nonetheless, *PMEL* is well conserved throughout vertebrate evolution, and we show the presence of purifying selection at the human *PMEL* locus. Given that our results support pigment cell-specific expression of PMEL, this suggests that PMEL performs an essential function in pigment cells.

This study provides a potential explanation why *PMEL* function is well conserved among vertebrates. Despite only a subtle effect of *Pmel* inactivation on visible pigmentation, we show a 40*–*50% reduction in eumelanin content in hair from *Pmel* knockout mice relative to controls. This demonstrates a substantially reduced efficiency in pigmentation, implying that PMEL likely has an essential function in vertebrates exposed to damaging UV light. This function might reflect a role for PMEL in making pigment production more effective by evenly distributing melanin polymerization throughout melanosomes. Such an enhancement of pigment efficiency might be most critical in the RPE, in which pigment formation is temporally limited to a brief pre- and post-natal period and in which persistent interactions with phagolysosomes might expose poorly polymerized melanins to degradative enzymes; indeed, a number of mutations in melanosome biogenesis appear to more severely affect pigmentation in the eye than in the skin [38]. Another possibility is that the polymerization of eumelanins on PMEL fibrils facilitates melanin transfer to keratinocytes, such that transfer is reduced in *Pmel* ^−/−^ skin.

The phenotype of the *Pmel* knockout mice is similar to the silvering phenotype described for the mouse *silver* allele that arose spontaneously [12*]*–[**14] and that is caused by a premature stop codon truncating the last 25 amino acid residues of PMEL [9], [10]. This allele dilutes eumelanin pigmentation but has no clear effect on pheomelanin pigmentation, consistent with the results in the present study. Furthermore, a previous analysis of melanin content showed that homozygosity for the *silver* allele caused a 20% reduction in eumelanin content on a recessive black (*Asip^a/a^*) background and 40% reduction on a brown (*Tyrp1^b/b^*) background [39], in good agreement with the present results for *Pmel* ^−/−^ mice (Table 1 and Table 2). The more pronounced effect of both *Pmel^si/si^* and *Pmel* ^−/−^ on eumelanin production on a brown background might reflect a combined effect of TYRP1 on tyrosinase stability [40], [41], facilitating melanin formation, and of PMEL on melanin polymerization/stability. However, a clear difference between *Pmel^si/si^* and *Pmel* ^−/−^ is that the *silver* allele shows a more pronounced dilution of pigmentation on a heterozygous brown background (*Tyrp1^b/+^*) than on the homozygous background (*Tyrp1^b/b^*) [12*]*–[**14], whereas we only observed a phenotypic effect in the homozygous brown background. This difference may be explained by the fact that the knockout is a null allele, while *silver* encodes a truncated form of PMEL that lacks the 25 C-terminal amino acids from the cytoplasmic domain. The *silver* PMEL variant is depleted but not absent from melanosomes [11], and thus might confer partial function that in some way is more deleterious in the presence of limiting amounts of functional Tyrp1.

Consistent with the influence of PMEL primarily on eumelanin accumulation shown here, the *Dominant white* allele in chickens [16] and the *Silver* allele in horses [22] both show a dominant inheritance and inhibit the production of eumelanin but have no visible effect on pheomelanin. Surprisingly, the *Dc* allele in cattle associated with a G22R missense mutation in *PMEL* is associated with a dilution of both pheomelanin and eumelanin, but further work is required to demonstrate the causative nature of the G22R mutation [25].

Our results suggest that the alleles described in other species with a more drastic effect on pigmentation, such as *Silver* in horses [22], *Merle* in dogs [24], and *Dominant white* in chicken [16] represent dominant negative mutations. The *Dominant white* and *Smoky* alleles in chickens provide a good illustration [16]. *Dominant white* inhibits the expression of black eumelanin and is caused by an insertion of three amino acid residues in the transmembrane domain. *Smoky* arose in a line homozygous for *Dominant white* and restored pigmentation. The causative mutation for *Smoky* is a deletion of four highly conserved residues in the PKD domain of PMEL. Thus, a plausible interpretation of these data, in relation to the phenotypic effects of the *Pmel* knockout mice, is that *Dominant white* is a dominant negative mutation while the *Smoky* mutation is a loss-of-function that inhibits the effect of *Dominant white*. Interestingly, we have recently demonstrated that the *Smoky* mutation indeed generates an essentially inactive form of PMEL, countering a dominant gain-of-function by the *Dominant white* mutation [28].

The present study now provides a reasonable explanation why only a single *Pmel* mutant has been described in mice compared with the fairly high number of mutations at most other pigmentation loci – that a *Pmel* loss-of-function has only a subtle effect on visible pigmentation. By contrast, dominant *PMEL* mutations in domestic animals are rather common; the present study explains why dominant-negative mutations have been selected in domestic animals without adverse pleiotropic effects in other tissues, since we confirm that PMEL is a melanocyte-specific protein. In this context, *Merle* dogs are unusual in having pleiotropic defects. The phenotypic effects associated with *Merle* in dogs is assumed to be caused by a retrotransposon insertion at the boundary of intron 10/exon 11 of *PMEL* [24]. In the heterozygous condition, *Merle* causes patches of diluted pigmentation and the effect is restricted to the inhibition of eumelanin pigmentation consistent with the effects of *Pmel* alleles in mice, chickens, and horses. *Merle* homozygotes show very pale pigmentation associated with hearing loss and visually defective microphtalmic eyes, and is considered sublethal due to multiple abnormalities of the skeletal, cardiac, and reproductive system [24], [42]. Given our results that loss of PMEL expression has only modest phenotypic effects, and that PMEL appears to be a melanocyte-specific protein, the broad phenotype observed in Merle dogs appears to be inconsistent with a loss-of-function mutation that affects only PMEL expression. There are several possible explanations for this enigma. Firstly, there are not yet any published data showing that *Merle* is associated with any altered expression of PMEL at the transcript or protein level or that expression of a wild-type form of PMEL can rescue the Merle phenotype. Thus, although it appears likely that *Merle* is indeed a *PMEL* mutant because the effect on pigmentation is restricted to eumelanin, as expected for a *PMEL* mutant, it cannot be formally excluded that the non-pigment effects of *Merle* reflect an additional mutation. However, the high rate of germ-cell reversions [42] is consistent with a single mutation caused by an insertion of a transposable element. Secondly, the severe effects on hearing and vision in *Merle* homozygotes compared with the apparently normal phenotype observed in *Pmel* knockout mice might be explained if *Merle* was the most severe dominant-negative mutation at the *Pmel* locus detected so far. Thus it might result in extensive melanocyte death in skin, eye, the inner ear, and even in the heart in which melanocyte loss is associated with abnormalities [43]. A third possible explanation for the pleiotropic effects on many different tissues in *Merle* dogs is potential ectopic expression of PMEL induced by the transposon insertion, and consequent toxic effects of amyloid formation outside the melanosome. Finally, since *PMEL* is located in a gene dense region (six genes within 100 kb) it is possible that the *Merle* mutation alters the regulation of more than one gene in the region. Thus, some of the phenotypic effects of *Merle* may be associated with PMEL function whereas others may be caused by altered expression of other closely linked genes.

An important justification for the development of knockout mice models is to suggest possible pathological effects of corresponding mutations in humans. The clear indication that purifying selection acts at the human *PMEL* locus suggests that loss-of-function mutations will cause reduced fitness. No human *PMEL* mutation associated with any phenotypic effects or disorders has yet been reported. Based on the results of the present study and previously described spontaneous mutations we can make the following predictions. Dominant-negative *PMEL* mutations, like *Dominant white* in chicken and *Silver* in horses, are expected to be associated with red hair and fair skin due to the expected reduction of dark eumelanin. However, *PMEL* variants do not appear to be a common cause of red hair and fair skin in humans [44]. Loss-of-function mutations must exist in the human population and no observable phenotypic effects are expected in heterozygotes. Loss-of-function homozygotes are expected on the basis of the phenotype of *Pmel* ^−/−^ mice to be fully viable but with a reduced content of eumelanin pigmentation in skin and hair. Thus, they are expected to be more sensitive to damaging UV-light and have a higher risk to develop various forms of skin cancer. *PMEL* has not been associated with an increased risk of skin cancer in genome-wide association studies (GWAS) in humans [45], but GWAS have a low power to identify rare genetic variants as expected for *PMEL* loss-of-function mutations.

Our observation of an altered shape of the melanosomes both in the RPE cells and in the uveal melanocytes of the eye suggests that *PMEL* ^−/−^ humans may also show eye disorders, perhaps in particular a higher incidence of age-related disorders such as macular degeneration. The ERGs did not reveal a drastic effect on retinal function, but since the sample size was small we cannot exclude a subtle effect. Furthermore, visual impairment does not always manifest in abnormal full-field ERGs. In this context it is worth noting that the *fading vision* mutant at the *PMEL* locus in zebrafish, in which a premature stop codon leads to production of a truncated protein, causes a severe developmental defect of the RPE and a consequent vision defect [23]. Furthermore, many Silver horses show an eye disorder named Mutiple Congenital Ocular Anomalities (MCOA) that resembles congenital aniridia in humans [46], [47]. Thus, the fact that three previously described *PMEL* mutations - *fading vision* in zebrafish, *Silver* in horses and *Merle* in dogs – are associated with different eye disorders and that the melanosomes in pigment cells in the eye of *Pmel* ^−/−^ mice have an altered shape clearly calls for further studies to explore if changes in PMEL function may lead to impaired vision in humans. The mouse model described here constitutes an excellent resource for such studies.

## Materials and Methods

### Gene targeting and construct design

The strategy for building the targeting construct using the pFlrt3-vector is shown in Figure 1A. A PCR-based strategy was employed by using a proofreading enzyme (KOD Hot Start DNA Polymerase, Novagen, USA). A pFlrt3-vector harboring two loxP sites, two FRT sites, and a neomycin cassette was used. A 5.0-kb upstream targeting arm starting in intron 1 (forward primer with Cfr42I tail and reverse primer with NotI tail, Table S2) and a 2.4-kb downstream targeting arm spanning from intron 3 to the beginning of intron 6 (forward primer with Bsp119I tail and reverse primer with XhoI tail, Table S2) were amplified. The 5.0-kb fragment was cloned into the Cfr42I and NotI sites while the 2.4-kb fragment was cloned into the BstBI and XhoI sites in the pFlrt3-vector. A 589 bp fragment spanning exon 2, intron 2, and exon 3 (forward primer with BglII tail and reverse primer also with BglII tail, Table S2) was cloned into the BglII site flanked by two loxP-sites.

According to the Ensembl genome assembly available at the time of the construct design, a knockout of the 589 bp fragment would cause a frame-shift between exon 1 and exon 4 leading to a premature stop codon and a non-functional PMEL protein. However, during the process of generating our transgenic mice, the Ensembl genome assembly was updated and the deletion of the 589 bp fragment turned out to cause an in-frame skipping of exon 2 and exon 3. Fortunately, the RNA was later shown to be degraded and the PMEL protein absent in the knockout mice (see Figure 1C and 1D).

The final target construct was linearized by XhoI and used for homologous recombination in R1 ES cells [48]. 408 clones were obtained and screened for homologous recombination by long range PCR. In total, 30 clones were revealed as positive for a targeting event. These were confirmed by Southern blot to be positive, using a probe targeting upstream of the 5.0-kb fragment (Sp5F and Sp5R, Table S2). Genomic DNA was extracted and digested with the restriction enzyme KpnI and separated by 0.7% agarose gel electrophoresis. Five clones were injected into blastocysts taken from a cross between C57BL/6NCrl females and B6D2F1/Crl males. The blastocysts were subsequently implanted into CD1 females. Two chimeric mice (crossed with C57BL/6J mice) were successfully used for germ line transmission. The F_1_ agouti offspring were confirmed to be heterozygous at the modified allele by PCR. F_1_ males were subsequently crossed with C57BL/6J PGK-Cre females in order to generate animals heterozygous for the null allele. After four generations of backcrossing into a C57BL/6J background, heterozygous animals were intercrossed to get homozygous null animals.

### Genotyping of mutant mice

PCR amplification of genomic DNA extracted from tail biopsies was performed to genotype mutant mice. Tail biopsies were incubated 45 min in 96°C in lysis buffer (250 mM NaOH and 2 mM EDTA) and the reaction was inhibited by the addition of 400 mM TRIS HCl pH 8.0. The offspring of the chimeric mice were genotyped for germline transmission using two primers amplifying the wild-type and the targeted allele (primer A in intron 1 and primer B in the loxP-flanked fragment, Table S2). Primers A and B generated a 236 bp band and a 392 bp band from the wild-type and the targeted allele, respectively (Figure 1A). The offspring of the PGK-Cre mice were genotyped to confirm the absence of the loxP-flanked fragment using three primers amplifying the lox and flox alleles (primers A, B, and D in the pFlrt3-vector flanked by the loxP and FRT sites, Table S2). Primers A and B amplified the floxed allele generating a 392 bp product, whilst no band was produced from the knocked allele. Primers A and D amplifying the floxed allele produced a 762 bp band and a 319 bp band was produced from the knocked allele.

### RNA isolation and qPCR analysis

RNA was isolated from skin tissue samples with the RNeasy mini kit (Qiagen). The RNA samples were subjected to reverse transcription using the cDNA high capacity kit (Applied Biosystems). mRNA transcripts for the two alleles were measured by quantitative PCR analysis using TaqMan Gene Expression master mix (Applied Biosystems) on a 7900HT Fast RT-PCR System (Applied Biosystems). Data were analyzed with a threshold set in the linear range of amplification, based on a standard curve of serial 10-fold dilutions for each primer set. The *Pmel* data were normalized using two endogenous housekeeping genes (*GAPDH* and *18S rRNA*) and plotted as fold change.

### Tissue preparation and immunohistochemistry

Whole eyes and skin were dissected and fixed in 4% PFA for 60 min, washed 10 min in PBS and cryoprotected in 30% sucrose for 3 and 12 h, respectively, before being frozen in OCT (Sakura). Tissues were cryosectioned (10 µm sections for eyes and 12 µm sections for skin) and collected on Superfrost Plus glasses (Menzel-Gläser). For immunohistochemistry, the sections were rehydrated in PBS for 15 min and then blocked in PBS containing 1% fetal calf serum, 0.02% thimerosal and 0.1% Triton X-100 for 30 min. Primary and secondary antibodies were diluted in this solution. Samples were incubated with primary antibodies overnight at 4°C, and with secondary antibodies for 2 h at room temperature. Samples were analyzed using a Zeiss Axioplan2 microscope, equipped with Axiovision software. Images were formatted, resized, enhanced and arranged for publication using Axiovision, and Adobe Photoshop.

### Antibodies

The following mouse monoclonal antibodies were used: HMB45 to PMEL (Lab Vision, Fremont, CA); TA99/Mel5 to TYRP1 (American Type Culture Collection, Manassas, VA; for immunofluorescence microscopy); and GTU-88 to γ-tubulin (Sigma-Aldrich, St. Louis, MO). Polyclonal rabbit antibodies included: H-90 to TYRP1 (Santa Cruz Biotechnologies, Santa Cruz, CA; for immunoblotting); Pep7 [49] to tyrosinase, αhPep13h [50] to the C-terminus of human PMEL, and αmPmel-N [11] to the N-terminus of mouse PMEL. Secondary antibodies included FITC labelled anti-rabbit immunoglobulin (Ig) antibodies (Vector laboratories) for immunofluorescence microscopy of eye tissues, or highly cross-absorbed goat or sheep antibodies to mouse Ig, rat Ig, or isotype-specific antibodies to mouse γ1 and γ2a heavy chains (Jackson Immunoresearch) conjugated to Alexafluor 488 or Alexafluor 594 (InVitroGen).

### Cell culture

Primary cultures of skin-derived melanocytes were generated as described [51]. Briefly, skins from neonatal wild-type C57BL/6 or *Pmel* ^−/−^ mice were digested with trypsin to separate the epidermis from the dermis, and the epidermis (containing the melanocytes) was further digested to a single cell suspension with trypsin and seeded onto a feeder layer of XB2 keratinocytes. Cells were cultured in RPMI 1640 (InVitrogen, Carlsbad, CA) supplemented with 10% FBS, 200 nM TPA, and 2 nM cholera toxin in a humidified incubator with 10% carbon dioxide. For analyses, cells were cultured without feeders.

### Immunoblotting—primary melanocytes

Cells were harvested with 5 mM EDTA in PBS, lysed with 0.5% SDS, 1% 2ME, and boiled to obtain whole cell lysates. Lysates corresponding to equivalent numbers of cells were fractionated by SDS-PAGE using Tris-glycine gels and then transferred to PVDF membranes (Millipore) for immunoblotting analysis as described [21], [50]. Protein bands were detected with alkaline phosphatase conjugated secondary antibodies, enhanced chemifluorescence, and phosphorimaging analysis using a Storm 860 fluorescence imaging system and ImageQUANT software (GE Biosciences).

### Immunoblotting—sclera

Eyes were collected from wild-type and *Pmel* ^−/−^ mice. The pigmented sclera was isolated by dissection and homogenized using a Tissue Tearor in lysis buffer (20mM Tris, 0.1% Triton, 0.1% SDS, 50mM NaCl and 2.5mM EDTA, 1nM Na_3_VO_4_ and 1X protease inhibitor cocktail (Roche)). 25µg of total protein lysates were denatured in sample buffer and reducing agent. The samples were separated on a bistris 8*–*12% gel (Invitrogen). Proteins were transferred to a hybond-C extra membrane (Amersham Biosciences). Unspecific binding to the membrane was blocked using 5% skimmed milk in TBS 0.1% Tween. Primary αPep13h or actin (Santa Cruz Biotechnology) antibody was added in blocking buffer in a 1∶1000 dilution O/N. The membranes were washed in TBS 0.1% Tween and HRP conjugated anti rabbit (GE Healthcare) or anti goat secondary (DAKO) antibodies in a 1∶4000 dilution. The membranes were subsequently washed in TBS 0.1% Tween and developed using ECL plus (GE Healthcare).

### Immunofluorescence and bright-field microscopy

Primary melanocytes were grown on matrigel-coated coverslips, fixed with 2% formaldehyde, labeled with primary and fluorochrome-conjugated secondary antibodies as described [21], [50], and analyzed on a DM IRBE microscope (Leica Microsystems, Wetzlar, Germany) equipped with an Orca digital camera (Hamamatsu, Bridgewater, NJ). Images were captured and manipulated using OpenLab software (Improvision, Lexington, MA) as described [52], and processed using Adobe Photoshop software.

### Electron microscopy

Primary melanocyte cultures were fixed in a mixture of 4% paraformaldehyde and 2% glutaraldehyde in cacodylate buffer pH 7.4, post fixed with 2% Osmium tetroxide harvested by scraping, dehydrated in ethanol, and embedded in epon resin. Ultrathin sections were contrasted with 2% uranyl acetate, analyzed by transmission electron microscopy on a Philips CM120 electron microscope (FEI, Eindoven, The Netherlands), and digital acquisitions were made with a numeric camera Keen View (Soft Imaging System, Munster, Germany). Individual melanosomes within electron micrographs of epon-embedded sections were identified and the maximum width and height of each melanosome was measured using OpenLab software. At least 200 melanosomes were counted for each cell type. Statistical analyses were done using Microsoft Excel linear regression analysis to determine the correlation coefficient between melanosome width and length.

Electron microscopy analysis of *stria vascularis* of the inner ear cochlea was done as follows. The mice were deeply anaesthetized with pentobarbital and transcardially perfused with 0.9% saline followed by fixative containing 2.5% glutaraldehyde and 0.5% paraformaldehyde in a phosphate buffer pH 7.2. After the animals were decapitated, their temporal bones were removed and their cochleas were dissected out. Local perfusion was carried out with the same fixative and the cochleas were left in the solution over night at +4^o^C. The cochleae were decalcified in 0.1 M EDTA for approximately one week until the bone was soft. Rinsing with phosphate buffer was performed several times followed by post fixation in 1% osmium tetroxide. The tissues were dehydrated and embedded in Agar 100 (Agar 100 Resin kit, Agar Scientific Limited). To orient the tissue, 1 µm thick sections were made on an ultratome (LKB Cryo Nova), stained with toluidine blue and investigated in a light microscope. The *stria vascularis* at the lower apical turn of the cochlea were chosen and ultra-thin sections were cut in this area, mounted on formvar coated copper grids, stained with uranyl acetate and lead citrate to be examined with a transmission electron microscope (JEOL 1230).

In the perfused animals the entire eyes were extirpated and transported to the morphology laboratory in the same fixative as the previous perfusion. The anterior segment of the eyes from each animal was discarded leaving posterior eyecups used for the light-and electron microscopic studies. Eyecups were incubated with gentle agitation in the same fixative for at least 2 h at room temperature. The full length of each eyecup was then gross sectioned with two vertical sections through the optic nerve head and 2 mm temporally. Samples were post-fixed in 1% osmium tetroxide and embedded in epoxy resin. They were washed with 0.17M sodium cacodylate, pH 7.4, followed by secondary fixation in 1% osmium tetroxide. Subsequently, the samples were dehydrated via sequential incubation in increasing concentrations of acetone and embedded in epoxy resin. Sections of the embedded samples were cut for both LM and EM examinations. For LM, 1 µm thick sections were mounted on glass slides and were stained with Toluidine blue. For EM, sections were mounted on copper grids and were stained with uranyl acetate and lead citrate. Light microscopy was performed using a Zeiss Axiophot microscope in order to choose areas of interest for the EM studies. The electron microscopy was performed using a JEOL 1200 EX transmission electron microscope.

### Electroretinography (ERG)

Two knockout mice (one male, one female, aged 23 months) and two age-matched mice were used for the experiment. The experiments were approved by the regional Ethical Committee and carried out following the guidelines of the ARVO guidelines for animal experimentation.

The ERGs were recorded under general anaesthesia induced by intraperitoneal injection of 3 mg/kg acepromazine (Plegicil vet 10 mg/ml, Pharmaxim Sweden AB, Alcon, Sweden), 100 mg/kg ketamine (Ketaminol vet 50 mg/ml, Intervet AB, Sollentuna, Sweden) and 20 mg/kg xylazine (Narcoxyl vet 20 mg/ml, Intervet AB, Sollentuna, Sweden).

The mice were placed in sternal recumbency. The ERGs were recorded using corneal electrodes (Goldring, 3 mm, Roland Consult, Brandenburg, Germany) with isotonic eye drops containing 0.2% hyaluronic acid (ZilkEye, Evolan Pharma AB, Danderyd, Sweden) as coupling agent. Subcutaneous platinum-iridium needle electrodes served as ground and reference electrodes (Technomed Subdermal EEG Needle Electrodes, Cephalon A/S, Nørresundby, Denmark).

Light stimulation was generated by a xenon strobe (Grass PS33+, Astro-Med Inc, West Warwick, RI, USA) and a diffuser on the inner surface of a Ganzfeld dome spread light. Neutral density filters (Kodak Wratten no. 96, Kodak Rochester, NY, USA) were used to decrease the stimulus intensity. Light intensity was measured by a light meter (IL-1700, International Lights, Peabody, MA, USA) at level with the tested eye in the Ganzfeld. Responses were amplified and filtered by a bandpass filter (0.1*–*1,000 Hz) and stored using a Powerlab system (Powerlab, SP8, ADInstruments (Europe) LTD, Chalgrove, UK).

The mice were dark-adapted overnight and anesthetized and prepared for ERG under dim red light. A heating pad was used to maintain the body temperature throughout the ERG. Dark-adapted responses were recorded over a 4.5 log unit range up to 3.0 cd/m^2^/s, starting with the dimmest stimulus. Four responses presented at a frequency of 0.05 Hz were averaged to each stimulus intensity. Cone responses were recorded after light adaptation in the Ganzfeld with a steady, white background light (25 cd/m^2^) for 10 min. Averaged cone transient responses were obtained in response to 3.0 cd/m^2^/s flashes presented at 1.1 Hz and finally a light adapted, 30 Hz, cone flicker response was recorded. Amplitudes and implicit times were determined for each response and measured according to convention.

### Three-generation intercross

Two *Pmel* ^−/−^ males (backcrossed four generations to C57BL/6 background) were intercrossed with ten BALB/C females to generate F_1_ offspring heterozygous at the *A* (*Agouti*), *B* (*Tyrp1*), *C* (*Tyrosinase*) and *Pmel* loci. Subsequently, ten F_1_ males and 20 F_1_ females were intercrossed to generate 147 F_2_ offspring, with segregating genotypes at the four loci. SNP markers were developed for the *Asip* (rs27342000), *Tyrp1* (rs32544046) and *Tyr* (rs31392322) loci (Table S2). The C57BL/6 (*Pmel* ^−/−^) and BALB/C mice were fixed for different alleles at these SNP positions. The F_2_ offspring were genotyped at the four loci using pyrosequencing (Biotage, Uppsala, Sweden). Oligonucleotides are listed in Table S1. The coat and skin color of the mice in the F_2_ generation were ocularly examined at four weeks of age.

### Assays of eumelanin, pheomelanin, and total melanin

Dorsal hairs of four-week-old mice were plucked and processed for chemical analysis of eumelanin to detect the specific degradation product, pyrrole-2,3,5-tricarboxylic acid (PTCA) upon alkaline hydrogen peroxide oxidation [33], and of pheomelanin to detect the specific degradation product, 4-amino-3-hydroxyphenylalanine (4-AHP) upon hydroiodic acid hydrolysis [36], as reported previously. Content of total melanin was determined as absorbance at 500 nm (A500) after solubilizing 1 mg hair in 900 µL Soluene-350 plus 100 µL water as previously described [34].

### Ethical statement

The appropriate local Swedish ethical committees have approved all experiments involving live animals (permissions C85/10 and C154/7).

## Supporting Information

Figure S1 Western blot analysis of the RPE and choroid layer lysates of *Pmel* ^−/−^ and wild-type mice. The lysates were fractionated by SDS-PAGE and analyzed by immunoblotting using the Pep13h antibody. Actin was used as a loading control. PMEL protein was detected in the wild-type lysate but not in the *Pmel* ^−/−^ lysate.
(TIF)

Figure S2 Sections of cochleas, dissected from the inner ear of *Pmel* ^−/−^ and wild-type mice, observed by electron microscopy. All cells, including the intermediate cells harboring the pigment are represented. The pigmentation was similar in the *Pmel* ^−/−^ samples (*c–f*) compared to wild-type (*a–b*).
(TIF)

Table S1 Amino acid identity between the mouse PMEL protein and PMEL proteins in other vertebrates (data from HomoloGene entry 5048, www.ncbi.nlm.nih.gov).
(DOCX)

Table S2 Oligonucleotides used for PCR and pyrosequencing analysis of the *Pmel* locus in mice. All three forward oligonucleotides used for pyrosequencing have an M13-tag sequence, allowing the M13-Biotin labeled oligonucleotide to anneal.
(DOCX)
