# Supplementary figures and images for: Inactivation of Pmel Alters Melanosome Shape But Has Only a Subtle Effect on Visible Pigmentation
Source: PLoS Genet. 2011 Sep 15;7(9):e1002285. doi: 10.1371/journal.pgen.1002285 (PMC3174228; doi:10.1371/journal.pgen.1002285)

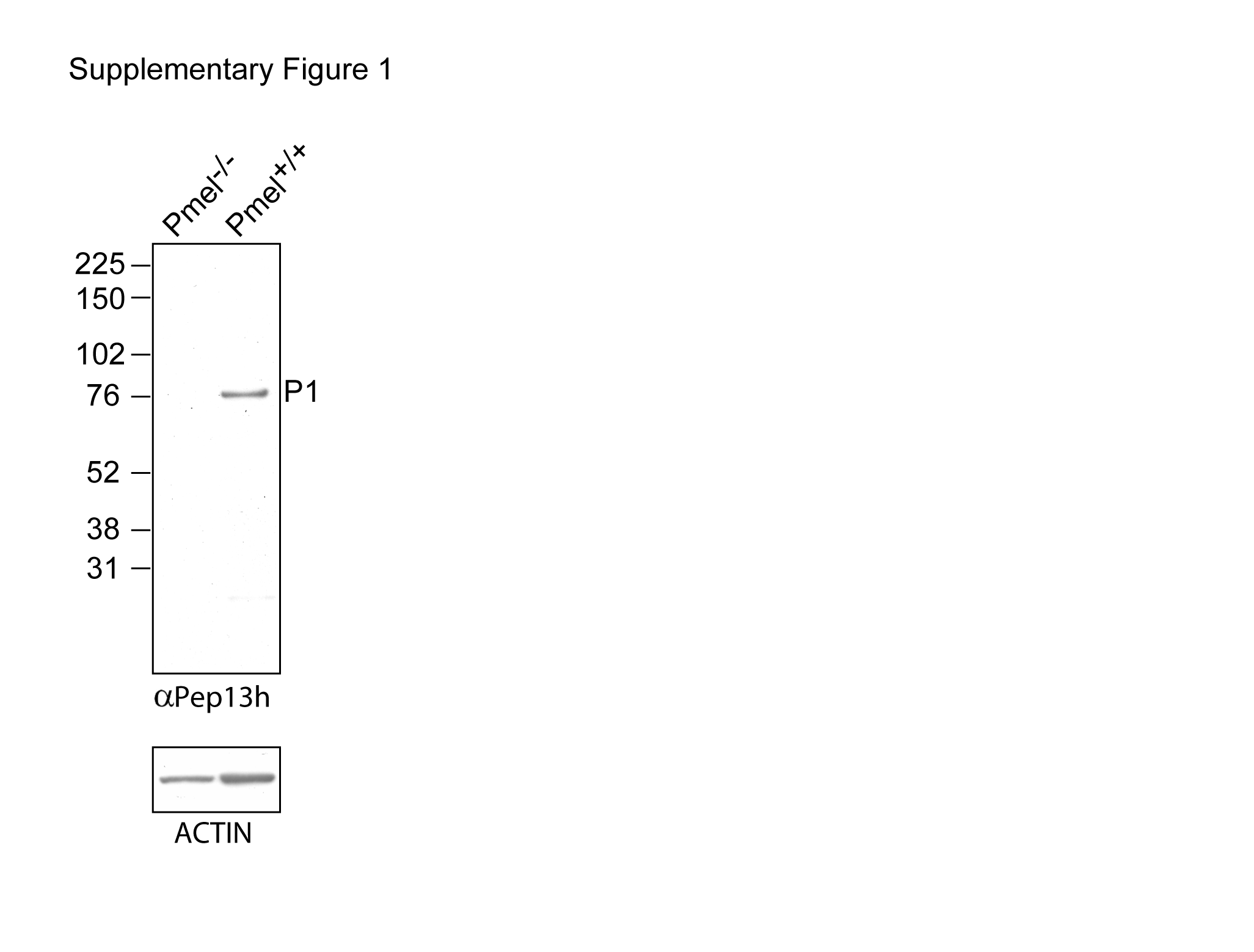

Supplement: Figure S1 — Western blot analysis of the RPE and choroid layer lysates of Pmel −/− and wild-type mice. The lysates were fractionated by SDS-PAGE and analyzed by immunoblotting using the Pep13h antibody. Actin was used as a loading control. PMEL protein was detected in the wild-type lysate but not in the Pmel −/− lysate. (TIF) [file pgen.1002285.s001.tif]

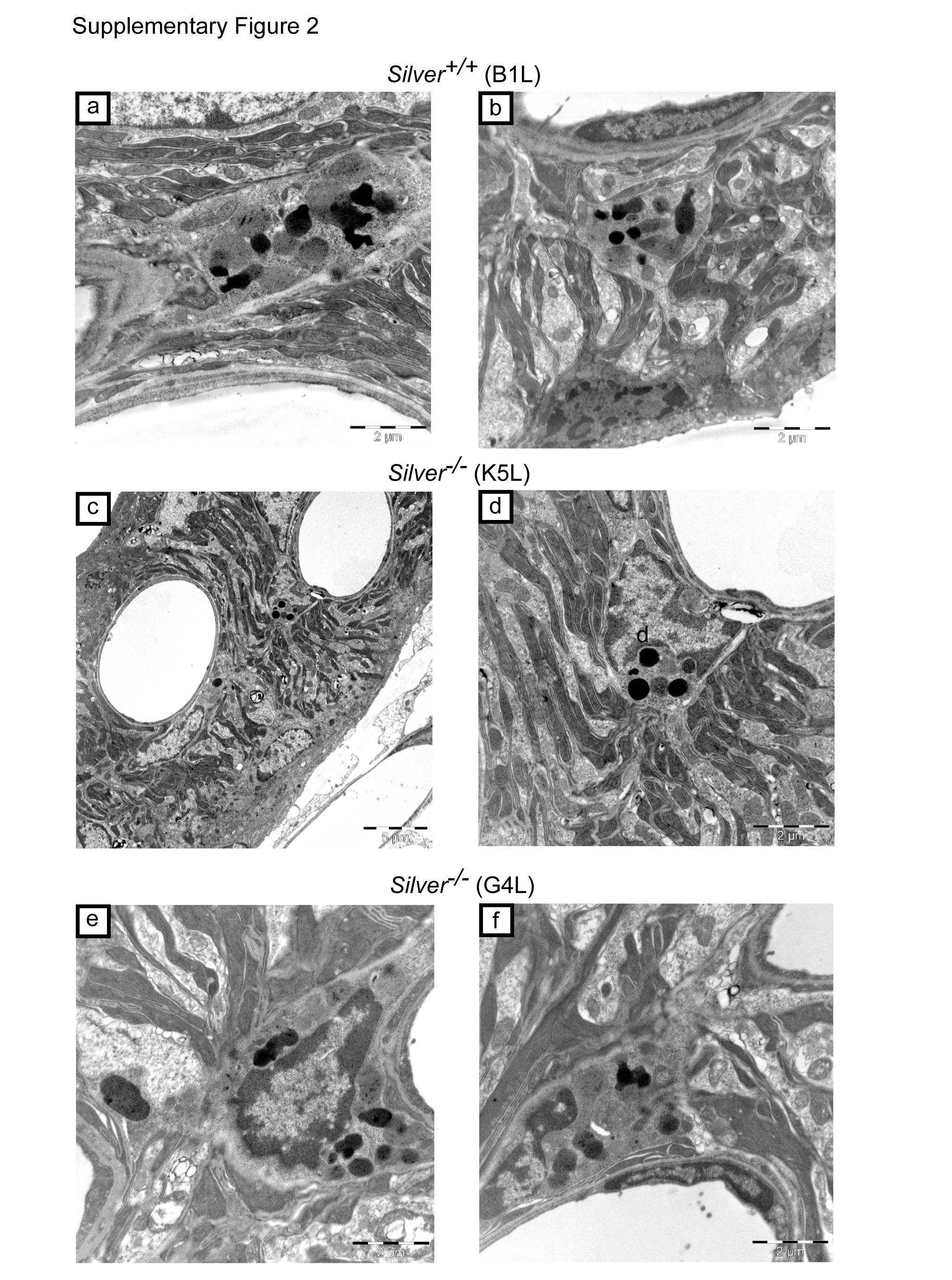

Supplement: Figure S2 — Sections of cochleas, dissected from the inner ear of Pmel −/− and wild-type mice, observed by electron microscopy. All cells, including the intermediate cells harboring the pigment are represented. The pigmentation was similar in the Pmel −/− samples (c–f) compared to wild-type (a–b). (TIF) [file pgen.1002285.s002.tif]
