# Supplementary material for: Inactivation of Pmel Alters Melanosome Shape But Has Only a Subtle Effect on Visible Pigmentation
Source: PLoS Genet. 2011 Sep 15;7(9):e1002285. doi: 10.1371/journal.pgen.1002285 (PMC3174228; doi:10.1371/journal.pgen.1002285)
Supplement: Table S1 — Amino acid identity between the mouse PMEL protein and PMEL proteins in other vertebrates (data from HomoloGene entry 5048, www.ncbi.nlm.nih.gov). (DOCX) [file pgen.1002285.s003.docx]

**Table S1**.

| Species | Amino acid identity (%) |
| --- | --- |
| Human | 79.8 |
| Chimpanzee | 80.0 |
| Cattle | 76.6 |
| Dog | 78.3 |
| Rat | 90.3 |
| Chicken | 47.4 |
| Zebrafish | 40.0 |
