# Supplementary material for: Inactivation of Pmel Alters Melanosome Shape But Has Only a Subtle Effect on Visible Pigmentation
Source: PLoS Genet. 2011 Sep 15;7(9):e1002285. doi: 10.1371/journal.pgen.1002285 (PMC3174228; doi:10.1371/journal.pgen.1002285)
Supplement: Table S2 — Oligonucleotides used for PCR and pyrosequencing analysis of the Pmel locus in mice. All three forward oligonucleotides used for pyrosequencing have an M13-tag sequence, allowing the M13-Biotin labeled oligonucleotide to anneal. (DOCX) [file pgen.1002285.s004.docx]

**Table S2.**

| **Oligonucleotide/**  **Amplicon** | **Forward primer** | **Reverse primer** |
| --- | --- | --- |
| PS_Tyrp1_rs32544046 | M13-CCACTTGTGACTGTATGCTCAGTACA | AGAGTATCAGGTGTCCTCTAGCACTC |
| PS_Agouti_rs27342000 | M13-GGTGAGGGAAGTGCTCTCAGATCAA | CCAGCTAGGTGGTACCTGCAATC |
| PS_Tyr_rs31392322 | M13-GCTGCTGTGGGAATATGGCTTGTT | GTTCTCTTCCACAGCATTAAGGGCT |
| M13-Biotin labeled | BIO-CACGACGTTGTAAAAC |  |
| Primer A | CCAAGTGTAGGCACCACTAT |  |
| Primer B | AGATCCTGGGATCCTTAGTC |  |
| Primer D | TTCCCACTCTAGAGGATCAG |  |
| Sp5 probe | GAATTCTCTTAAATG CAGTGATAGGAGA | GAATTCCTGAAG CAAGAACAGTCATAAG |
| 5.0 kb fragment | GAATTACCGCGGCTAACCTGGGATGCAGAGATAGAGAAGGAG | CCCGCGGCCGCATTAATTTTGGCGGTATGGAAAGCAGCACT |
| 589 bp fragment | CCCAGATCTAAATTTCACTTTGAATTTAACAGTGAGATGAC | CCCAGATCTTTAAAAAATAGAAGAGGGAGGGGACTTTCC |
| 2.4 kb fragment | GAATTATTCGAAACAGTGCTGGGGTTCAAACTTAGGGCCTCA | CCCCTCGAGTGGCAGGATCAGGGTGTTAGAATCTGACATATCTG |
